# COVID-19 outbreak in a state prison: a case study on the implementation of key public health recommendations for containment and prevention

**DOI:** 10.1186/s12889-022-12997-1

**Published:** 2022-05-14

**Authors:** Catherine Duarte, Drew B. Cameron, Ada T. Kwan, Stefano M. Bertozzi, Brie A. Williams, Sandra I. McCoy

**Affiliations:** 1 Division of Epidemiology, School of Public Health, University of California, Berkeley, 2121 Berkeley Way West, Berkeley, CA 94704 USA; 2 Department of Health Policy and Management, Yale School of Public Health, Yale University, 60 College St, New Haven, CT 06510 USA; 3 Division of Health Policy and Management, School of Public Health, University of California, Berkeley, 2121 Berkeley Way West, Berkeley, CA 94704 USA; 4 Division of Pulmonary and Critical Care Medicine, University of California, San Francisco, 1001 Potrero Ave, San Francisco, CA 94110 USA; 5 School of Public Health, University of Washington, 1959 NE Pacific St, Seattle, WA 98195 USA; 6 Instituto Nacional de Salud Pública, Universidad No. 655, Cuernavaca, MOR 62100 México; 7 The UCSF Center for Vulnerable Populations, University of California, San Francisco, 2789 25th St, San Francisco, CA 94110 USA

**Keywords:** COVID-19, Prisons, Decarceration, Policy, Vaccination, Case study, Conceptual framework

## Abstract

**Background:**

People incarcerated in US prisons have been disproportionately harmed by the COVID-19 pandemic. That prisons are such efficient superspreading environments can be attributed to several known factors: small, communal facilities where people are confined for prolonged periods of time; poor ventilation; a lack of non-punitive areas for quarantine/medical isolation; and staggeringly high numbers of people experiencing incarceration, among others. While health organizations have issued guidance on preventing and mitigating COVID-19 infection in carceral settings, little is known about if, when, and how recommendations have been implemented. We examined factors contributing to containment of one of the first California prison COVID-19 outbreaks and remaining vulnerabilities using an adapted multi-level determinants framework to systematically assess infectious disease risk in carceral settings.

**Methods:**

Case study employing administrative data; observation; and informal discussions with: people incarcerated at the prison, staff, and county public health officials.

**Results:**

Outbreak mitigation efforts were characterized by pre-planning (e.g., designation of ventilated, single-occupancy quarantine) and a quickly mobilized inter-institutional response that facilitated systematic, voluntary rapid testing. However, several systemic- and institutional-level vulnerabilities were unaddressed hindering efforts and posing significant risk for future outbreaks, including insufficient decarceration, continued inter-facility transfers, incomplete staff cohorting, and incompatibility between built environment features (e.g., dense living conditions) and public health recommendations.

**Conclusions:**

Our adapted framework facilitates systematically assessing prison-based infectious disease outbreaks and multi-level interventions. We find implementing some recommended public health strategies may have contributed to outbreak containment. However, even with a rapidly mobilized, inter-institutional response, failure to decarcerate created an overreliance on chance conditions. This left the facility vulnerable to future catastrophic outbreaks and may render standard public health strategies - including the introduction of effective vaccines - insufficient to prevent or contain those outbreaks.

**Supplementary Information:**

The online version contains supplementary material available at 10.1186/s12889-022-12997-1.

## Background

People incarcerated in US prisons have been disproportionately harmed by the COVID-19 pandemic [1, 2]. Between March and June 2020, the COVID-19 case rate was 5.5 times higher among people incarcerated in US prisons than in the general population [3]. By August, 90% of the largest COVID-19 clusters nationally were in prisons and jails [4]. That carceral settings are such efficient superspreading environments can be attributed to a number of known factors. The physical space is characterized by small, communal facilities where people are confined for prolonged periods of time, poor ventilation, aging infrastructure, and a dearth of appropriate, non-punitive areas for quarantine and medical isolation [1, 2, 5–8]. Carceral facilities are also extremely porous, with staff commuting daily from surrounding communities and incarcerated people regularly transferred between and within jails, prisons, and detention centers [1, 2, 5, 9]. In the presence of a highly contagious, respiratory pathogen, these circumstances - combined with staggeringly high numbers of people experiencing incarceration - essentially assure the occurrence of superspreading events [10, 11]. Further, owing both to pre-incarceration structural determinants (e.g., poverty, racism, medical marginalization) and the health harms of incarceration itself, people incarcerated in US prisons have disproportionately high rates of chronic medical conditions placing them at greater risk for severe COVID-19 complications and death [5, 10, 12].

Given these factors, several health organizations have issued guidance on preventing COVID-19 infection and mitigating spread in carceral settings [2, 4–6, 9, 13–16]. However, to-date only one study has documented the extent to – and process by – which recommendations have been implemented [1]. In April and May of 2020, a COVID-19 outbreak occurred at a state prison (hereafter “Prison A”) in California’s central coast region – one of 35 adult prisons in the California Department of Corrections and Rehabilitation (CDCR). The outbreak, one of the first in a California prison, culminated in 14 people testing positive before containment. In June 2020, the Office of the Federal Receiver overseeing healthcare in most California state prisons requested we evaluate how the outbreak was contained with so few cases to inform future mitigation strategies. We were guided by the following overarching questions:

1. What factors facilitated containment?
2. Were those factors a function of planning, responsiveness, and/or chance?
3. Do vulnerabilities to COVID-19 outbreaks remain?
4. What might prevent future outbreaks at Prison A?
5. What lessons might be transferable to other carceral settings or translated into policy?

We present our findings using an adapted public health framework describing the confluence of characteristics unique to carceral settings that shape risk for COVID-19 outbreaks. We then identify critical next steps given persistent and catastrophic outbreaks affecting Prison A in the months that followed and that continue to affect carceral facilities throughout CDCR and across the country.

## Methods

This manuscript presents our methodology and observations from the rapid evaluation of a COVID-19 outbreak response requested by the Office of California’s Federal Receiver overseeing the state’s prison healthcare system. Our goal was to provide timely information that could set priorities and inform urgent COVID-19 prevention and containment. Our investigation drew on the following sources: a June 10, 2020 informal meeting with 3 county public health officials; observation of the Prison A facility (e.g., cells and dormitories, “respiratory isolation unit,” medical facilities, yards, vocational shops) during a June 11, 2020 site visit; a meeting with approximately 10 Prison A officials, informal discussions with Prison A staff members; and informal discussions – overseen by Prison A officials – with people incarcerated at Prison A (including meetings with approximately 10 people incarcerated at Prison A who were members of various prison-based organizations selected by Prison A officials) during the site visit; administrative data provided by the County Public Health Department and CDCR (e.g., clinical and sociodemographic data); and publicly available data via CDCR web portals (e.g., COVID-19 case numbers, occupancy data). Meetings and informal discussions did not rely on formal interview guides and were neither recorded nor transcribed for analysis. Under Federal Regulations 21 CFR 50.3 and 45 CFR 46.102, this rapid evaluation is considered a quality improvement activity for the purpose of bringing about immediate improvement in a local setting and was not deemed “human subjects research ”[17].

### Conceptual Framework for Evaluating Risk of COVID-19 Outbreaks in Carceral Settings

**Fig. 1:**
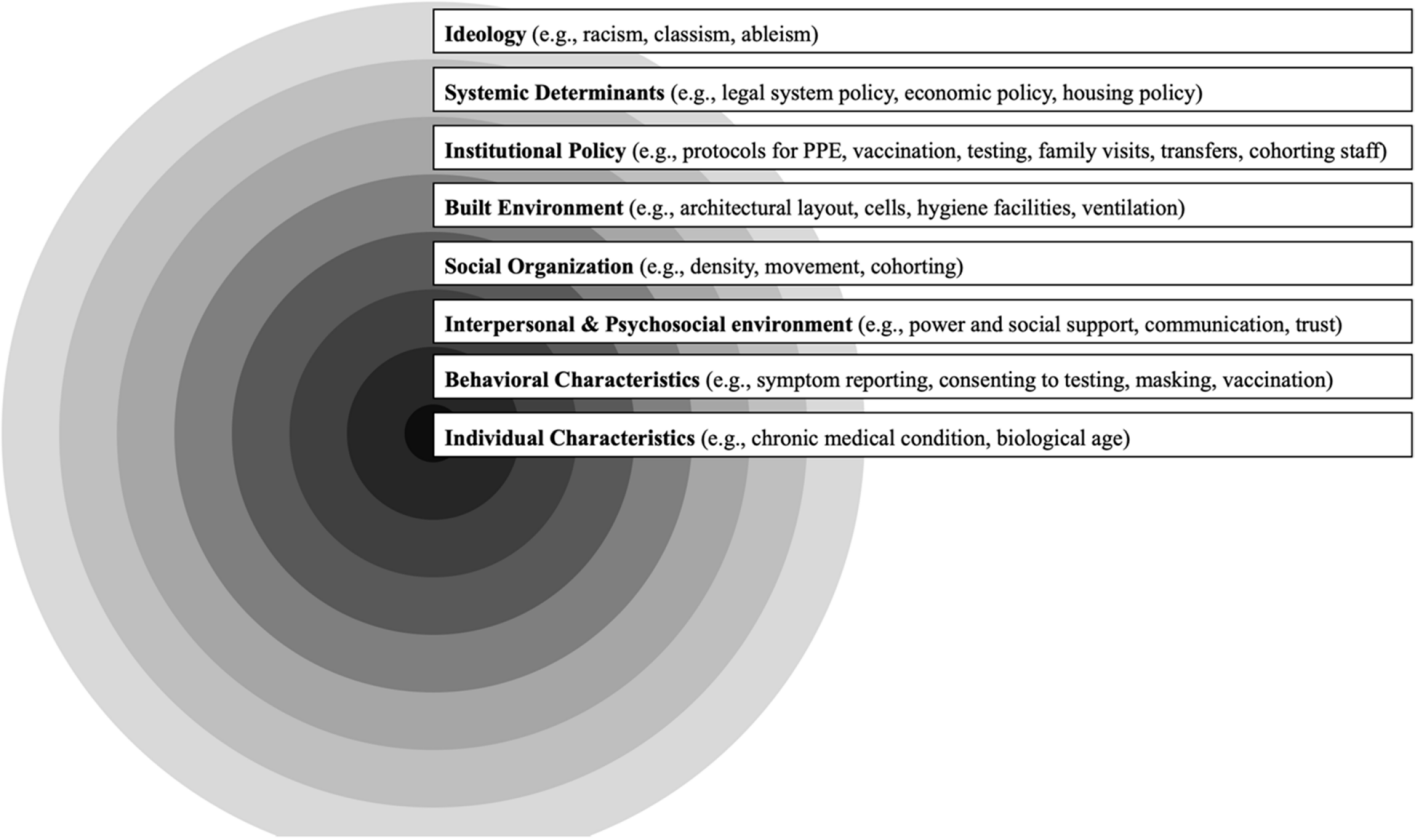
Ecosystem of COVID-19 Infection Determinants and Risk Factors in Carceral Settings. Note. Figure presents an adapted social determinants of health framework illustrating determinants and risk factors for COVID-19 and other infectious diseases operating at multiple levels within and beyond carceral facilities. PPE = personal protective equipment. Source. Adapted from Dahlgren, G. and Whitehead, M. (1991). Policies and Strategies to Promote Social Equity in Health. Stockholm, Sweden: Institute for Futures Studies and Neeley, A.N., et al., Building the Transdisciplinary Resistance Collective for Research and Policy: Implications for Dismantling Structural Racism as a Determinant of Health Inequity. Ethnicity & Disease, 2020. 30(3): p. 381

Building from existing models, we developed a framework to describe the multi-level system of determinants and risk factors that combine to influence risk for COVID-19 outbreaks in carceral settings (Fig. 1) [18, 19]. This conceptual framework illustrates how individual (micro) characteristics are nested within and thereby interact with each of the increasingly outer layers to shape distributions of COVID-19 infection within and beyond carceral settings. This extends to ideological and systemic (macro) determinants (e.g., racism, legal policy) that shape the likelihood of COVID-19 outbreaks as well as the inequitable risk of incarceration itself. We organize our analysis according to this framework, providing a specific description of each of the framework’s layers in the corresponding Results subsection.

## Results

Prison A is comprised of two complexes: East Facility (est. 1961) and West Facility (est. 1954) [20]. On March 1, 2020, there were 3,782 people incarcerated at Prison A (approximately 99% of physical design capacity). During the April-May 2020 outbreak, the case rate for all people incarcerated throughout the CDCR system was similar to the California population (~0.5 confirmed cases per 1000 people) and less than the US population (1.5 per 1000) [21]. In the county surrounding Prison A, daily incident COVID-19 cases were also low (never exceeding 13 new cases per day) [22]. The first positive test at Prison A was recorded on April 11. That sample was collected the previous day from an individual transferred to Prison A on April 6 from a southern California jail. In total, 14 cases were reported before containment – 11 among incarcerated people and three among staff. One additional officer assigned to West (dormitory units) was on leave when they tested positive. The outbreak was considered officially contained on June 9, 2020, when the final person who had tested positive received their second negative test result under the serial testing protocol.

### Ideology

As illustrated by their positioning as the most macro-level in the conceptual framework (Fig. 1), ‘ideologies’ – like racism, classism, and ableism – lead to and reinforce systemic determinants, influence institutional policies, and thereby create downstream consequences. This results both in the disproportionate targeting of marginalized populations for incarceration as well as their differential retention in carceral settings [5, 23]. Evidencing these effects, structurally marginalized people are over-represented in carceral settings throughout the US, [5] including in CDCR (among people incarcerated throughout CDCR, 29% identify as Black, 45% as Latinx, 21% as White, 1% as Asian, and 1% as Native American; whereas in the overall California population, 6% identify as Black, 40% as Latinx, 36% as White, 17% as Asian, and 1% as Native American) [24]. Among people incarcerated at Prison A at the time of the outbreak, 44% identified as Black, 19% Latinx, 19% White, and 18% “other” [25]. Approximately 9% had an Americans with Disabilities Act (ADA)-classified disability [25]. Prison A staff (*N*=1,719) identified as White (54%), Latinx (30%), “other” (12%), or Black (4%). Given that carceral settings are particularly efficient COVID-19 transmission environments, these circumstances may contribute to inequitable distributions of COVID-19-related harm [26].

### Systemic determinants

Comprising the second layer of the conceptual framework, ‘systemic determinants’ (e.g., legal policy, economic policy, housing policy) are factors that collectively shape the likelihood of incarceration, and population density in carceral settings, which in turn have implications for COVID-19 prevention and mitigation (Fig. 1). Throughout the COVID-19 pandemic, public health experts have joined calls for decarceration as the single most important strategy to prevent and contain COVID-19 in carceral settings [5, 14]. On March 31, 2020, CDCR announced it would begin decarcerating people who were due for release within 60 days and not currently incarcerated for charges of “violent crime as defined by law, domestic violence, or a person required to register under Penal Code 290” [27]. CDCR estimated that in an initial round 3,500 of the state’s 120,414 incarcerated people would be released (2.9% of total population) [27]. These releases to parole were completed on April 13 without further action announced until July 10 [27].

### Institutional policy

The third layer of the conceptual framework, ‘institutional policy’ (e.g., CDCR and/or Prison A-specific institutional protocols around testing, availability of personal protective equipment [PPE], transfers between facilities) can amplify or hinder efforts to stem COVID-19 transmission. In March, CDCR announced the suspension of transfers into prisons – affecting roughly 3,000 people incarcerated in California jails [28]. Other institutional policies implemented at this time included: increased availability of hygiene products (e.g., soap, hand sanitizer); suspension of visitation, entry of volunteers, and many rehabilitative program providers; and restriction of within-facility movement among incarcerated people [28]. CDCR also reported distribution of healthcare guidelines - based on the Centers for Disease Control and Prevention (CDC) and California Department of Public Health’s recommendations for carceral settings – to staff and incarcerated people regarding “infection control, assessment, testing, treatment, proper use of PPE and quarantine/isolation,” [28] though these practices were not mandatory. Lastly, CDCR modified “the delivery of non-emergent health care procedures such as routine dental cleanings to redirect supplies of PPE” [28] though subsequent international guidance on COVID-19 in carceral settings has advised against disrupting baseline healthcare services [2, 16]. To note, at the time, vaccines were not yet available.

The Prison A outbreak took place shortly after CDCR announced its new transfer policy. While the policy reduced the total number of people transferred, it did not halt all between-facility transfers, resulting in the first positive test (Additional file 1). At Prison A, CDCR policies to suspend programming and restrict the movement of incarcerated people prompted the introduction of staggered mealtimes, initiation of some cell-side services (e.g., commissary), and the discontinuation of outside recreation “yard” time. While people incarcerated at Prison A were cohorted (i.e., grouping people to limit transmission), mandating officer cohorting was not possible due to union contracts. Daily symptom screening and temperature checks started on March 14. Prison A hygiene protocols, including frequent surface disinfection, were also established in March and sanitation products were made available to staff and incarcerated people. While PPE use was encouraged, Prison A Medical’s requests to CDCR for PPE were initially unmet due to a systemwide shortage (N95 masks were obtained from a nearby paint store). In addition to low supply, PPE fit and quality were variable.

Prison A’s policies around medical isolation and quarantine were already established when the first known case of COVID-19 entered the facility. Given prior experience with norovirus, varicella, and influenza outbreaks at Prison A, and COVID-19 outbreaks in nearby carceral facilities, a “respiratory isolation unit” was designated. This unit, which did not rely on restrictive housing commonly used for punitive isolation (“solitary confinement”), comprised a set of solid-door, single-occupancy cells on a closed tier with shared multi-person showers (described further in ‘Built Environment’ section). The person transferred from the southern California jail was quarantined in this unit upon arrival while awaiting test results.

The county public health department contacted Prison A Medical after being notified of the first positive case through state-mandated morbidity reporting. When people incarcerated on a separate floor in the same building developed symptoms consistent with COVID-19, standardized outbreak investigation procedures were led by the county public health department – which also provided test kits due to CDCR shortages (at the time of the outbreak, Prison A was equipped with 15 and it was unclear as to when additional test kits would be made available through CDCR). In coordination with Prison A Medical, this included contact tracing and “concentric” testing of people confined to cells at increasing distance from the index case. The 11 positive cases identified through this process were moved to the “respiratory isolation unit” and underwent serial testing (at regular intervals until at least two sequential negative tests). It was also determined that an officer working in the outbreak building had entered a neighboring building, resulting in testing all 400 people incarcerated in both buildings and approximately 200 staff. Instead of using the existing CDCR laboratory contractor, these samples were analyzed by the county’s public health department laboratories, with significantly faster turnaround times averaging 24 hours (range: 0-4 days). Subsequent tests were administered to those who had initially tested negative. During this time, the index outbreak building was placed on lockdown for approximately three weeks, characterized by confinement to single cells without access to outdoor recreation "yard" time, while staff were permitted to enter and exit the unit.

### Built environment

The ‘built environment’ layer of the framework describes how any given prison’s physical infrastructure (e.g., architectural layout, ventilation systems) influences risk for COVID-19 transmission by imposing physical restrictions on decision-making at the more micro-levels of the framework (Fig. 1) [1]. For example, elements of the built environment such as facility design capacity and shared airspace modify COVID-19 risk associated with population density, like rebreathing virus-laden air. Notably, by nesting the ‘built environment’ layer at the meso-level, the conceptual framework also models how decision making at the more macro-levels (e.g., decarceration policies) likely shape the magnitude of influence that the built environment can have on the more micro-levels. For example, absent efforts to decarcerate, the built environment’s role in COVID-19 transmission would likely balloon to the point of rendering mitigation efforts (e.g., symptom reporting) ineffective as the superspreader risk associated with population density collided with restrictions imposed by the physical infrastructure (e.g., a dearth of non-punitive quarantine/isolation units) [1]. Conversely, significantly reducing the prison population through release could offset many key limitations of the built environment. However, unless the incarcerated population went to zero via release, the built environment would likely remain of concern for COVID-19 transmission via pathways like shared airspace.

Prison A’s East and West Facilities are architecturally different. West consists of 1940s-era military barracks, converted to open, shared airspace dormitories, each containing single or bunked beds and one shared toilet and shower facility (designed to account for approximately 37% of Prison A’s total capacity) [20]. East is divided into four independent yards and a stand-alone, 50-bed mental health crisis unit [20]. These quadrangles are lined by two 3-story buildings with solid-door, single-occupant cells on closed tiers, sharing multi-person showers (designed to account for approximately 63% of Prison A’s total capacity) [20]. The unit to isolate positive cases was located in one of these East buildings. Given these structural conditions, the outbreak occurring on East (single cells) and not West (dormitory units) likely facilitated containment. Additionally, the outbreak occurred in warmer weather when windows were open and heating systems were inactive, better diffusing any virus-laden air.

### Social organization

In our conceptual framework, we use the language of ‘social organization’ to indicate the effects of the group-level dynamics themselves on COVID-19 transmission, including density, movement, and cohorting among staff and people who are incarcerated (Fig. 1) [1]. These group-level dynamics both influence the effects of elements at the conceptual framework’s more micro-levels (e.g., density and cohorting shaping risk for COVID-19 infection and adverse outcomes among incarcerated people who are older or with chronic medical conditions) and are themselves influenced by the framework’s more macro-levels (e.g., systemic determinants like decarceration policy or institutional policy around cohorting staff).

At Prison A, ground markers to guide physical distancing were added in areas where people congregated to receive services, like daily medication distribution. With a focus on preventing droplet spread, dormitories we observed in West – which housed between 30-50 people – were subdivided into ‘pods’ (of 8 bunked beds) separated by 6 feet. Mask wearing was not required within ‘pods.’ Given a single shared airspace, however, the threat of widespread transmission via aerosols remained.

In East facility, people were incarcerated in single-occupancy cells. Despite union contract barriers to mandating staff cohorting, most staff elected to remain within units. However, as previously noted, at least one officer who later tested positive moved between buildings. Additionally, while most staff resided within 30 miles of Prison A, some commuted more than 140 miles, often by van-share, and some stayed at nearby hotels during shift days (Additional file 2).

### Interpersonal and psychosocial environment

The ‘Interpersonal and Psychosocial Environment’ layer of the framework centers the importance of (1) the nature of interactions between individuals and (2) the mental, emotional, and social context – as they both relate to power, social support, communication, trust - for COVID-19-related adversity and COVID-19 transmission risk (Fig. 1) [1].

In informal conversations with Prison A staff, some reported feeling overworked, while others feared contracting COVID-19 which resulted in high levels of medical leave. In informal discussions with people incarcerated at Prison A, they reported feelings of fear, anxiety, and mistrust particularly due to limited personal agency to implement prevention recommendations (e.g., accessing sanitation products to adhere to institutional policies around hygiene protocols, requesting officers adhere to PPE guidelines, reducing housing density). They also described how suspension of visits and loss of privileges (such as time out of cell) typically associated with punitive measures were mental health harming. These adverse experiences were compounded by communication lapses (e.g., people incarcerated in the unit locked-down during the outbreak reported receiving no communication from administration for that 3-week period). Finally, people incarcerated at Prison A remarked that the resources for psychosocial wellbeing during this time were profoundly insufficient. These reflections were consistent with what Prison A staff described as the impossibility of meeting regular health care needs in addition to addressing the urgency of an outbreak.

### Behavioral characteristics

The ‘behavioral characteristics’ layer of the conceptual framework refers to health-related decisions that individuals make (e.g., reporting symptoms, consenting to testing, masking, vaccination), as influenced by conditions at each of the macro-levels within which it is nested (Fig. 1). For example, institutional policies around medical isolation/quarantine in solitary confinement cells can influence willingness among incarcerated people to report symptoms [1]. Similarly, institutional policies encouraging proper PPE use that are differentially imposed on incarcerated people compared to staff can influence interpersonal power dynamics and disrupt collective efficacy around COVID-19 prevention and mitigation.

To medically isolate symptomatic cases, Prison A did not rely on restrictive housing commonly used for punitive isolation (“solitary confinement”). This choice likely reduced barriers to symptom reporting that have been experienced in other carceral facilities and may have contributed to outbreak containment [29, 30]. By contrast, discussions with staff revealed that, while aware of mitigation measures to prevent fomite and droplet transmission, some staff reported low risk perceptions for COVID-19 introduction and onward transmission within Prison A. This may have been influenced by the differential imposition of institutional guidelines for staff, as described by people incarcerated at Prison A, with implications for staff guideline adherence. For example, while proper PPE use was encouraged at the institutional-level, use was inconsistent among staff. Further, during the outbreak, there was greater initial testing refusal among staff compared to people incarcerated at Prison A, with even greater re-testing refusal among staff (~50%).

### Individual characteristics

Finally, as the most micro-level of the conceptual framework, individual characteristics – like living with a chronic medical condition or being of older age – are nested within and thereby interact with each of the increasingly outer layers to shape distributions of COVID-19 infection and adverse outcomes (Fig. 1). This indicates that the magnitude of risk associated with any individual characteristic is dependent on decisions at all other levels (e.g., systemic decisions to decarcerate; institutional policy around testing, vaccination, or staff cohorting).

Compared to CDCR averages, people incarcerated at Prison A are older (25% vs. 38% age 50+, respectively) and have higher prevalence of chronic conditions: 34% have at least one medical condition associated with increased risk of severe complications from COVID-19;13% have four or more. Among Prison A employees, 33% are age 50 or older.

## Discussion

In our evaluation of the first COVID-19 outbreak at Prison A, we found evidence that people incarcerated at the prison, several staff, and the county public health department planned ahead and mobilized rapidly to contain the outbreak. Yet using our public health-focused conceptual framework, we also identified several factors that could have hindered containment and posed unattended risks for future, larger outbreaks. These factors included CDCR’s inconsistent policy of halting transfers between facilities; delays in PPE and test kit provision; union regulations preventing staff cohorting; inconsistent staff use of PPE; and an overreliance on both (1) verbal symptom screening and temperature checks rather than rapid testing and (2) stemming fomite and droplet spread rather than aerosolized spread. Several of these factors reflect a COVID-19 knowledge base that was still evolving in the scientific community and the general public in the initial months of the pandemic. However, one established critical strategy that was not meaningfully implemented was decarceration.

### Containment: planning, responsiveness, and/or chance?

Several pre-planning and response actions taken likely contributed to outbreak containment. These included the inter-institutionally coordinated response between county public health and Prison A Medical, which facilitated rapid, systematic, and voluntary testing in units with a COVID-19 exposure, quick turnaround of test results through the county’s public health laboratories, and the early designation of a well-ventilated, single-occupancy isolation unit [1]. Still, several chance events played a favorable role in containment, portending worse future outbreaks: (1) the officer who tested positive was on leave, likely sparing the dorms from a COVID-19 introduction; (2) instead, the outbreak occurred in East where conditions (e.g., physical infrastructure, less densely populated) created a lower probability of onward spread; (3) there was only one active COVID-19 case among people transferred into Prison A resulting in fewer demands on limited isolation units; (4) despite barriers to cohorting officers, spread between buildings was limited as most staff elected consistent assignments in the same units; and (5) COVID-19 prevalence in the surrounding county was low, limiting the likelihood of multiple, simultaneous introductions by staff from the community [2, 22].

Indeed, following the April-May 2020 outbreak, Prison A experienced persistent and increasingly larger outbreaks. At the peak of a subsequent outbreak on January 13, 2021, there were 1,195 active cases (39% of the incarcerated population) – the highest within CDCR – contributing to Prison A’s inclusion on a list of carceral facilities across the country with the highest cumulative case counts [21, 31, 32]

### Public health implications

Given existing guidance for COVID-19 prevention and control in carceral settings, our recommendations focus on strategies that have been insufficiently implemented in carceral settings throughout the US and/or areas where existing guidance falls short [4–6, 9, 13–16]. We organize these recommendations according to Lopez et al.’s Hierarchy of Controls [9]. While recognizing the necessity of each intervention, this framework emphasizes how, absent efforts to fundamentally shift conditions through decarceration, carceral settings render insufficient what are typically effective public health interventions for containing infectious disease outbreaks [1]. Notably, the majority of interventions employed at Prison A were classified at the lower levels of the Hierarchy of Controls rather than those at higher, more impactful levels (e.g., elimination-level). As persistent outbreaks threaten Prison A and other carceral facilities throughout the US, we provide justification for prioritizing the below recommendations, while guidance for their optimal and simultaneous implementation can be found in Table 1.

**Table 1:** Application of the Hierarchy of Controls to Strategies for the Prevention and Mitigation of COVID-19 Transmission in Carceral Settings

**Recommendation**	**Implementation Guidance**	**Hierarchy of Controls**
Prioritize the health, wellbeing, and dignity of incarcerated persons	• Support the physical and mental health needs of people who are incarcerated by maintaining all existing healthcare services without interruption and addressing the risk of health harms caused by the imposition of further restrictive measures and loss of privileges from COVID-19 mitigation measures ◦ Particularly during the pandemic, meeting federal/international mandates regarding healthcare provision in carceral settings relies on the urgent implementation of decarceration strategies • Communicate clearly, effectively, honestly, and non-coercively through trusted avenues with people who are currently incarcerated and their loved ones ◦ Consult people who are currently and formerly incarcerated, as well as their families, in co-developing and implementing prevention and intervention approaches	Overarching
Urgently decarcerate	• Let public health imperatives (e.g., physical distancing requirements, minimizing shared airspace, maintaining existing healthcare services, and meeting new care demands) guide minimum requirements for population reduction. • Do not rely on transfers to meet population reduction targets as transfers between facilities to relieve crowding at one institution necessarily increases density at another and therefore transmission risk. • Include support for reentry (e.g., housing, health care access) through investments in and collaborations with existing non-carceral, community-led reentry services. • Monitor and report number of people decarcerated at the institution-level in addition to system-wide	1: Elimination
Prioritize people who are incarcerated and staff for vaccination	• Couple vaccination with decarceration to maximize individual and population health • Given the uniquely hazardous risk that carceral settings – and staff movement within, to, and from carceral settings – pose, implement mandatory vaccination requirement as a condition of employment for custody and staff • Make vaccination universally available to people who are incarcerated and obtain their informed, free from coercion consent to be vaccinated	2: Substitution
Maximize air exchange in all indoor facilities	• Minimize potential for rebreathing air through reductions in population density • Categorize population density on the basis of individuals within a common airspace - not based upon potentially porous physical barriers like walls and doors that may be circumvented by heating and cooling systems. • Maximize opportunities for time spent outdoors to reduce the accumulation of virus-laden aerosols • Consult external HVAC (Heating, ventilation and air conditioning) experts to evaluate unique facility characteristics: expert determination is necessary to ensure ventilation, air exchange, and air filtration systems meet recommendations for airborne infectious aerosol exposure established by the American Society of Heating; Refrigerating; and Air Conditioning Engineers (ASHRAE)	3: Engineering controls
Limit density of housing units	• Coordinate strategies to limit the size and density of housing units through decarceration • Prioritize the use of single-occupancy units with closed doors *and* adequate ventilation (not recirculated air) whenever possible, especially for individuals with multiple underlying conditions that increase risk for adverse COVID-19 outcomes • Prioritize reducing the occupancy of large dorms, reserving them for group isolation of people who have tested positive for SARS-CoV-2	4: Administrative controls
Employ rapid testing, screening, and epidemiologic surveillance of staff and incarcerated people	• Urgently decarcerate facilities with support for re-entry to maximize the effectiveness of testing, screening, and epidemiologic surveillance efforts • Ensure that system-wide procedures include systematic and voluntary: (1) d*iagnostic* testing of symptomatic individuals (with turnaround times ≤24 hours for results); (2) frequent testing of exposed individuals; (3) widespread screening of staff and incarcerated people • Align widespread screening frequency with transmission risks and disease prevalence in surrounding communities • For prisons with particularly low prevalence, ongoing pooled testing can minimize burden and increase rapid outbreak detection	4: Administrative controls
Prioritize prevention and control measures among staff	• Urgently decarcerate facilities with support for re-entry to minimize risks associated with staff introductions of SARS-CoV-2. • Ensure proper and consistent use of PPE and provision of standard N95 masks (without one-way valves) – including frequent replacement with new masks or effective disinfection of used masks – is facilitated for staff and people who are incarcerated • Designate locations for the quarantine and medical isolation of staff in order to protect incarcerated people, families of staff, and the surrounding community • Negotiate with union representatives and state agencies in charge of staffing procedures to facilitate proper cohorting of staff within and between facilities • Given the outsized risk posed to the safety and wellbeing of incarcerated people and surrounding community, implement protocols for frequent testing and mandatory vaccination among staff	4: Administrative controls 5: PPE

Notes. Each recommendation (Column 1) is accompanied by guidance on key considerations for optimal implementation (Column 2). The recommendations are listed according to the Lopez et al. Hierarchy of Controls framework (Column 3). This framework, adapted specifically for carceral settings, emphasizes how known COVID-19 control and prevention strategies situated at the lower end of the Hierarchy of Controls are dependent on those at the higher end to be successful, therefore facilitating the prioritization of strategies.

Sources. Lopez, W.D., et al., Preventing the Spread of COVID-19 in Immigration Detention Centers Requires the Release of Detainees. American Journal of Public Health, 2020(0): p. e1-e5.; UNODC, WHO, UNAIDS and OHCHR joint statement on COVID-19 in prisons and other closed settings. 2020 [cited 2021 January 26]; Available from: https://www.who.int/news/item/13-05-2020-unodc-who-unaids-and-ohchr-joint-statement-on-covid-19-in-prisons-and-other-closed-settings.; ASHRAE Epidemic Task Force. [n.d.] “Core Recommendations for Reducing Airborne Infectious Aerosol Exposure” Available from: https://www.ashrae.org/file%20library/technical%20resources/covid-19/core-recommendations-for-reducing-airborne-infectious-aerosol-exposure.pdf; ASHRAE Epidemic Task Force. 20 October 2020. “Building Readiness.” Available from: https://www.ashrae.org/file%20library/technical%20resources/covid-19/ashrae-building-readiness.pdf; Science Brief: SARS-CoV-2 and Potential Airborne Transmission. 2020 [cited 2021 March 16]; Available from: https://www.cdc.gov/coronavirus/2019-ncov/more/scientific-brief-sars-cov-2.html.; Preparedness, prevention, and control of COVID-19 in prisons and other places of detention: interim guidance. Copenhagen: WHO Regional Office for Europe, 2021, February 8. Available from: https://www.euro.who.int/en/health-topics/health-determinants/prisons-and-health/publications/2021/preparedness,-prevention-and-control-of-covid-19-in-prisons-and-other-places-of-detention-interim-guidance,-8-february-2021-produced-by-whoeurope

#### Prioritize the health, wellbeing, and dignity of people incarcerated in jails, prisons, and detention centers

Prioritize the health, wellbeing, and dignity of people incarcerated in jails, prisons, and detention centers through support for emotional and psychological needs; continuous, honest, and non-coercive communication via trusted avenues; and consultation in co-developing and implementing prevention and intervention approaches. Especially given the risk of harm from enacting additionally restrictive measures in environments where individuals are already deprived of liberty, [2] the successful and ethical implementation of all other recommendations depends on a clear and consistent commitment to this overarching priority [1, 16].

#### Urgently decarcerate to reduce population density

The single most effective strategy to prevent and limit COVID-19 spread is to dramatically decrease population density at each prison through decarceration [2–6, 9, 11, 14]. Reductions in population density are central to the feasibility and effectiveness of every public health recommendation [15]. Furthermore, decarceration cannot be achieved through transfers to other carceral settings (e.g., prisons, jails, detention centers) as transfers to relieve crowding at one institution necessarily increase density at another and therefore transmission risk. Rather, decarceration must be explicitly defined as large-scale releases of people from confinement with support for optimal community reentry through investments in and collaborations with non-carceral, community-led reentry services [5, 11].

#### Prioritize people who are incarcerated and staff for vaccination

With the uniquely hazardous risk that carceral settings pose, people in these environments should receive high priority status for vaccination. While necessary, however, vaccination is insufficient in carceral settings – particularly given evidence of diminished effectiveness in preventing spread in densely populated settings with high transmission rates; histories of abuse and harm against people who are incarcerated and refusals among staff (e.g., union efforts in California to circumvent vaccine mandates for staff which create additional risks for incarcerated populations who do not have the individual agency to opt out of this high transmission risk environment), creating barriers to achieving requisite vaccination thresholds for stemming transmission; waning immunity, and emerging variants that could bypass or reduce the effectiveness of current vaccines [11]. Thus, vaccination (ideally mandatory vaccination for staff and strongly encouraged for people who are incarcerated) must be coupled with other strategies, particularly decarceration, to maximize individual and population health.

#### Maximize air exchange in all indoor facilities

Aerosolized spread is a common transmission pathway in carceral settings given enclosed conditions, prolonged exposure to respiratory particles, and inadequate ventilation – further exacerbated during lockdowns and winter months [33, 34]. Therefore, minimizing rebreathing air through significant reductions in population density (i.e., to limit the number of people in a shared airspace), permitting yard time, and consultation with heating, ventilation, and air conditioning experts knowledgeable about reducing risk of airborne transmission of infectious diseases are all essential to reducing COVID-19 transmission risk.

#### Limit density of housing units

**Fig. 2:**
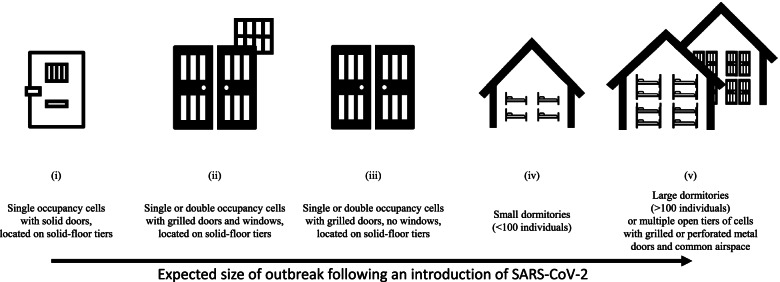
One dimension of COVID-19 risk in carceral facilities: potential risk of larger outbreaks increases with more densely populated units and larger shared airspaces. Note. Whereas ‘facility design capacity’ is an architectural definition that does not have salience for risk of COVID-19 infection (i.e., a prison can be below design capacity and still pose an insurmountable superspreader risk absent decarceration), risk of larger outbreaks increases with more densely populated units and larger shared airspace. Risk of infection increases with the number and proportion of positive cases. This figure does not account for other known transmission routes apart from the unit, which can drastically change risk for onward transmission (e.g., via ventilation systems that may recirculate rebreathed air laden with virus)

Prisons are highly heterogeneous. Consequently, each structure poses unique challenges for COVID-19 prevention and control efforts. Housing units vary considerably in risk for transmission, with dormitories and large, multi-level units with shared airspace presenting a particularly high risk of respiratory disease spread through aerosolized particles (Fig. 2). As such, a prison can be below physical design capacity and still pose an insurmountable superspreader risk if these heterogenous housing units comprise a larger shared airspace. Limiting unit-specific density through decarceration is essential—especially in the highest risk housing units and workspaces.

#### Employ rapid testing, screening, and epidemiologic surveillance of staff and people who are incarcerated

Frequent, voluntary testing is necessary for successful outbreak response. This includes diagnostic testing of symptomatic individuals, frequent testing of exposed individuals, widespread screening of staff and people who are incarcerated with frequency tied to community prevalence, and non-punitive responses to provide care for those who test positive.

#### Adopt prevention and control measures among staff

Because of movement within and between facilities and communities, staff are most likely to introduce SARS-CoV-2 into prisons [35]. This outsized risk posed to the safety and wellbeing of incarcerated people must be addressed through adequate masking, frequent testing, mandatory vaccination (for example, as is required of most hospital employees), and cohorting of staff.

## Conclusion

The COVID-19 pandemic finds us positioned at the intersection of decades of unprecedented incarceration and a global pandemic unlike any seen in a lifetime. After May 2020, Prison A experienced several more COVID-19 outbreaks as have all other California prisons [21]. At its worst thus far, the COVID-19 case rate for people incarcerated in CDCR was 10.4 times that of the California population and 12.7 times that in the US population – a far cry from spring 2020 conditions [21]. Our observations evidence the unique and dynamic susceptibility of carceral settings to respiratory pathogens, which may be further tested with vaccine-resistant variants, vaccination refusals among prison staff, or in the next respiratory pandemic. Our analysis suggests that even with rapidly mobilized and coordinated efforts among incarcerated people, staff, and public health officials, containment in these high-risk, congregate settings can still be largely determined by chance, a point underscored by subsequent, large-scale outbreaks at Prison A and other prisons. These findings support existing calls for decarceration as the cornerstone for addressing respiratory pandemics in prisons, one that must be implemented immediately so that its protective effects precede – therefore preventing or maximizing efforts to contain – outbreaks. To address COVID-19 and protect the public’s health, action must be grounded in evidence – not dependent on chance.

## Supplementary Information

**Additional file 1. **Incoming transfers and prevalent active COVID-19 cases among incarcerated persons.

**Additional file 2. **Zip codes from which Staff Commute to Prison A.

## Data Availability

Publicly available documents are accessible through the California Department of Corrections and Rehabilitation webpage [https://www.cdcr.ca.gov/]. Observational data described in the manuscript were collected by authors: AK, CD, SB, SM. Remaining data that support the findings of this study from the California Department of Corrections and Rehabilitation are not publicly available but are available from the authors with permission.
