# Supplementary material for: COVID-19 outbreak in a state prison: a case study on the implementation of key public health recommendations for containment and prevention
Source: BMC Public Health. 2022 May 14;22:977. doi: 10.1186/s12889-022-12997-1 (PMC9107313; doi:10.1186/s12889-022-12997-1)
Supplement: Supplementary file 1 — Additional file 1. Incoming transfers and prevalent active COVID-19 cases among incarcerated persons. [file 12889_2022_12997_MOESM1_ESM.docx]

**Appendix A. Incoming transfers and prevalent active COVID-19 cases among incarcerated persons**

***
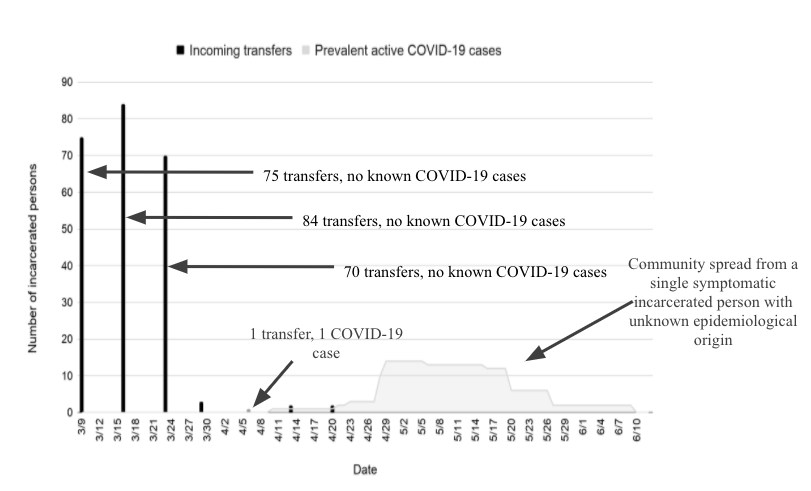
***

***Note.*** *CDCR policy to halt transfers was implemented in March 2020. Blue bars represent incoming transfers to Prison A from other jails and prisons. Shaded region illustrates known daily point prevalence of active COVID-19 cases.*

***Note.*** *Total number of positive COVID-19 cases can be impacted by several factors, including testing turnaround time, people being transferred from other jails and prisons, people being transferred within a prison (e.g., East to West at Prison A), and onward transmission in the prison. For example, the longer the testing turnaround time, the longer quarantined individuals must remain under observation, and the greater the daily point prevalence.* *Testing turnaround of approximately 24 hours facilitated reductions in daily point prevalence as resolved COVID-19 cases were released from conditions of quarantine. There were no other new introductions at this time.*

***Sources:*** *San Luis Obispo County Department of Public Health; California Department of Corrections and Rehabilitation*
