# Supplementary material for: COVID-19 outbreak in a state prison: a case study on the implementation of key public health recommendations for containment and prevention
Source: BMC Public Health. 2022 May 14;22:977. doi: 10.1186/s12889-022-12997-1 (PMC9107313; doi:10.1186/s12889-022-12997-1)
Supplement: Supplementary file 2 — Additional file 2. Zip codes from which Staff Commute to Prison A. [file 12889_2022_12997_MOESM2_ESM.docx]

**Appendix B. Zip codes from which Staff Commute to Prison A**
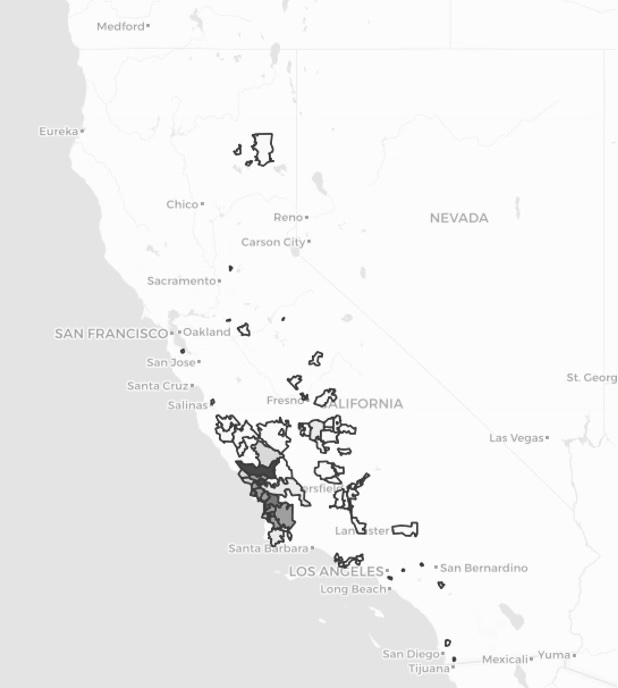

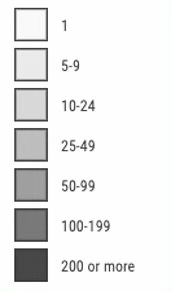


***Note.*** *Concentration of Prison A staff by zip code of residence*

***Source.*** *California Department of Corrections and Rehabilitation*
